# Connective tissue disease-related interstitial lung disease is alleviated by tripterine through inhibition of the PI3K/Akt, apoptosis, and TNF-α signalling pathways

**DOI:** 10.3389/fphar.2022.990760

**Published:** 2022-11-07

**Authors:** Wen Zhu, Yifan Wang, Chenxi Liu, Yunxia Wu, Yehui Li, Yue Wang

**Affiliations:** ^1^ Institute of Rheumatology, Nanjing University of Chinese Medicine, Nanjing, China; ^2^ Jiangsu Province Hospital of Chinese Medicine, Nanjing, China; ^3^ Department of Rheumatology, Affiliated Hospital of Nanjing University of Chinese Medicine, Nanjing, China

**Keywords:** tripterine, CTD-ILD, network pharmacology, molecular docking, PI3K/AKT, apoptosis

## Abstract

**Background:** Interstitial lung disease (ILD) is the major cause of morbidity and mortality in patients with various rheumatic diseases. However, more interventions need to be sought. Tripterine, an extract of *Tripterygium wilfordii* Hook. F, has been widely studied for its powerful anti-inflammatory effect. However, its mechanism of action in treating connective tissue disease-related (CTD)-ILD remains unclear.

**Purpose:** To investigate the mechanism of tripterine in CTD-ILD treatment by combining network pharmacology and an *in vivo* experiment.

**Methods:** The related targets of tripterine were obtained after searching the Traditional Chinese Medicine System Pharmacology Database and Analysis Platform, Comparative Toxicogenomics Database, GeneCards, Search Tool for Interacting Chemicals database, and SymMap database. Following this, Online Mendelian Inheritance in Man, GeneCards, Genebank, and DrugBank were used to screen the targets of CTD-ILD. A target-signalling pathway network was constructed using Cytoscape. Additionally, topological analysis was performed. Protein interaction analysis was performed using the STRING online analysis platform. Following this, Gene Ontology (GO) and the Kyoto Encyclopaedia of Genes and Genomes (KEGG) signalling pathway enrichment analyses were performed. Subsequently, the molecular docking between tripterine and the core targets was verified. Finally, experimental verification was performed in bleomycin-induced model mice.

**Results:** A total of 134 common targets and 10 core targets of tripterine, including signal transducer and activator of transcription 3, tumour necrosis factor (TNF), v-rel avian reticuloendotheliosis viral oncogene homolog A, protein kinase B (Akt) *α* (Akt1), mitogen-activated protein kinase (MAPK) 1, Jun transcription factor family, tumour protein 53, MAPK3, nuclear factor kappa B subunit 1, and caspase 8, were obtained. GO enrichment analysis revealed that, while treating CTD-ILD, tripterine was mainly involved in cytokine receptor binding, receptor-ligand activity, signal receptor activation, cytokine activity, protein ubiquitination, deoxyribonucleic acid transcriptase activity, etc. The KEGG pathway enrichment analysis revealed that the most significant signalling pathways were multiple viral infections and the phosphatidylinositol-3-kinase (PI3K)/Akt, TNF, and apoptosis signalling pathways. Molecular docking results revealed that tripterine had good docking activity with the core targets. Experimental studies also demonstrated that tripterine could inhibit the activation of PI3K/Akt, apoptosis, and TNF-α signalling pathways in lung tissue and significantly improve lung pathology and collagen deposition in the model mice.

**Conclusions:** This study preliminarily revealed the potential molecular biological mechanism of tripterine while treating CTD-ILD might be related to inhibiting the PI3K/Akt, apoptosis, and TNF-α signalling pathways. *Tripterygium wilfordii* Hook. F. and its extract could be used clinically for treating CTD-ILD.

## Introduction

Connective tissue disease (CTD), a common clinical autoimmune disease involving multiple organs and systems, usually affects the respiratory system. Interstitial lung disease (ILD) is one of its severe pulmonary complications (Spagnolo et al., 2021). Studies have indicated that approximately 40% of patients with ILD also have CTD (Mira-Avendano et al., 2019). By far, the aetiology and pathogenesis of CTD-related ILD (CTD-ILD) remain unclear; however, immune-mediated lung inflammation and subsequent fibrosis are the key steps (Spagnolo et al., 2021). Infiltration of inflammatory cells in the lung interstitium is thought to be responsible for the early stages of ILD. Disease progression causes collagen deposition and fibrocyte proliferation, resulting in the pathological manifestations of fibrosis in the advanced stage (Desai et al., 2018). Regulating various pro-inflammatory factors and restoring immune homeostasis primarily inhibits the progression from inflammation to fibrosis in this disease (Hoffmann-Vold et al., 2019). Therefore, in recent years, glucocorticoids combined with immunosuppressive agents have been mainly administered for clinically treating CTD-ILD. It is noteworthy that numerous immunosuppressants used for treating CTD-ILD have varying degrees of adverse reactions and limited efficacy (Gao and Moua, 2020).

Recently, it has been reported that transforming growth factor (TGF)-β1 (Celada et al., 2018; O'Leary et al., 2020), platelet-derived growth factor (PDGF) (Zhu et al., 2019), vascular endothelial growth factor (VEGF), and fibroblast growth factor (FGF) play a vital role in pulmonary fibrous tissue proliferation through their effects on leukocytes and angiogenesis (Chaudhary et al., 2007). A popular drug for treating ILD, Nintedanib, plays an antifibrotic role by targeting the above molecular mechanisms (Hoffmann-Vold et al., 2019); however, it is expensive and has several side effects. Hence, it may not be the drug of choice for patients. Other interventions, such as complementary therapies, are yet to be explored.

Our previous study reported that *Tripterygium wilfordii* Hook. F. polyglycoside tablets satisfactorily treated CTD-ILD; however, its mechanism of action remains unclear (Li et al., 2021). Tripterine is a natural pentacyclic triterpenoid compound extracted from the root bark of *Tripterygium wilfordii* Hook. F. It has good anti-inflammatory, antioxidant, antitumour, immunosuppressive, and neuroprotective biological properties (Chen et al., 2018). Animal experiments have confirmed that tripterine has a significant therapeutic effect on injured lung tissue (He et al., 2021); however, most studies were mainly about rheumatic diseases, and research on its applicability for treating CTD-ILD and its molecular biological mechanism are scarce. Therefore, this study aimed to study the mechanism of tripterine, which is anticipated to be effective in treating CTD-ILD.

**FIGURE 1:**
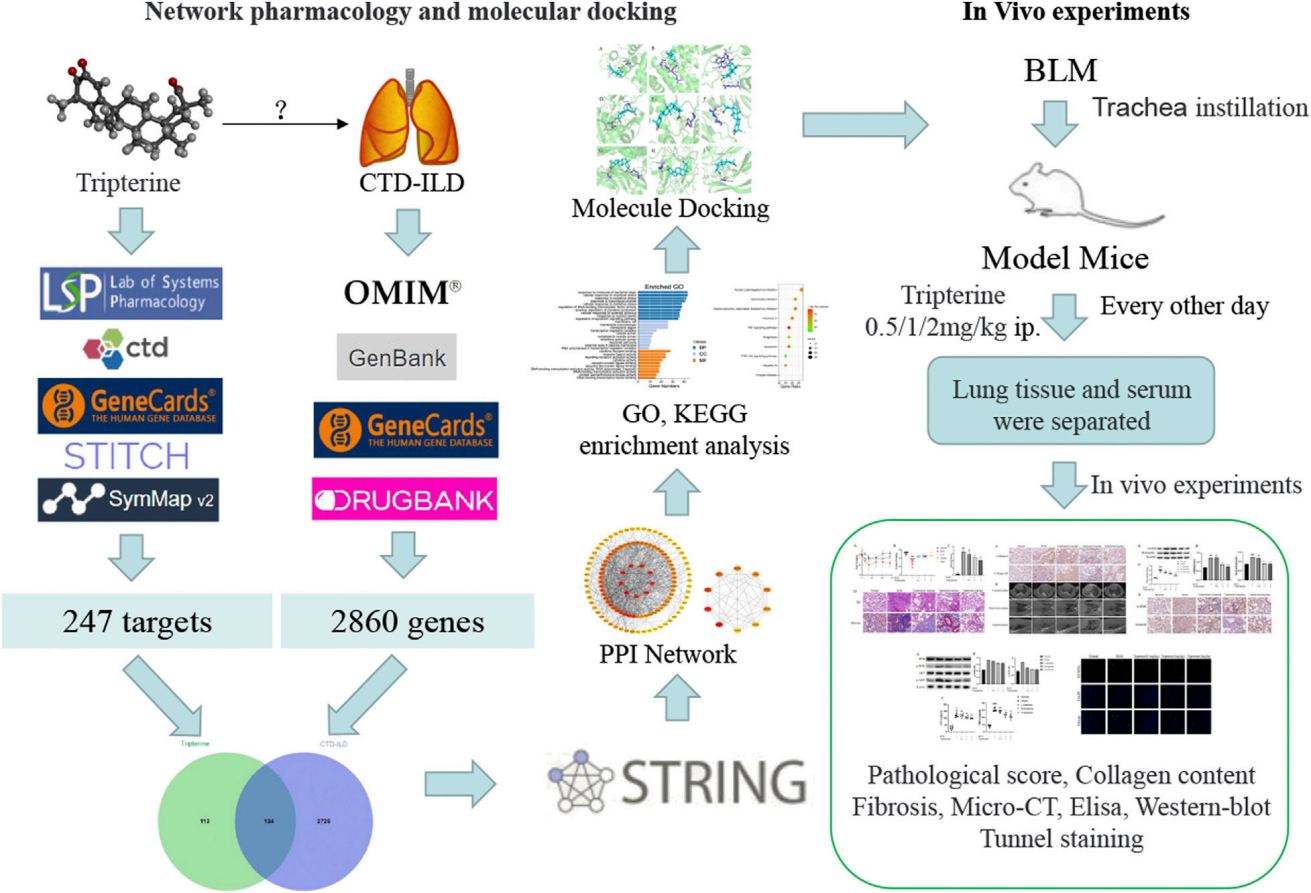
Workflow of the network pharmacology analysis and experimental verification in this study.

Network pharmacology, a comprehensive research method based on a disease-gene-drug action target network (Yang et al., 2013), provides a fresh perspective for studying the complex interaction between potential traditional Chinese medicine components and disease targets. Nevertheless, the predicted results need to be experimentally verified. Therefore, the network pharmacology method was adopted in this study to explore the molecular mechanism of tripterine in the treatment of CTD-ILD. Following this, the results were verified using molecular docking technology and *in vivo* experiments. The concrete research workflow is illustrated in Figure 1.

## Materials and methods

### Screening of related targets

The eight databases exploited to obtain the related targets were as follows: the Traditional Chinese Medicine System Pharmacology Database and Analysis Platform (TCMSP) (http://tcmspw.com/tcmsp.Php), Comparative Toxicogenomics Database, (http://ctdbase.org/), GeneCards database platform (https://www.genecards.org), Search Tool for Interacting Chemicals (STITCH) database (http://stitch.embl.de/), SymMap database (https://www.symmap.org/), Genebank database (https://www.ncbi.nlm.nih.gov/genebank), Online Mendelian Inheritance in Man (OMIM) (http://www.omim.org), and DrugBank database (https://www.drugbank.ca). ‘Tripterine’ was used as the keyword to retrieve key targets from the TCMSP, Comparative Toxicogenomics Database, GeneCards, STITCH, and SymMap databases. “Connective Tissue Disease-related Interstitial Lung Diseases” was then used as the retrieval term in OMIM, GeneCards, Genebank, and DrugBank. The potential targets were obtained after eliminating the duplicated targets. Organism equal to *Homo sapiens* was limited.

### Intersection target acquisition and protein-protein interaction (PPI) network construction

R software (R 4.0.2) was used to map tripterine potential targets to CTD-ILD-related targets, and a Venn diagram was drawn to obtain an intersection of the two targets. The targets common to tripterine and CTD-ILD were uploaded to the STRING database (https://string-db.org), the species was selected “*Homo sapiens*,” and the confidence was set at > 0.9. A PPI network was constructed by importing the results into Cytoscape 3.8.0.

### Gene Ontology (GO) and the Kyoto Encyclopaedia of Genes and Genomes (KEGG) enrichment analysis

R software (R 4.0.2) was used to analyse the molecular function (MF), biological process (BP), and cellular components (CC) to identify the interaction characteristics of the common targets in the gene function and signalling pathway. The KEGG pathway enrichment analysis was performed to analyse the possible tripterine interventional pathway in CTD-ILD.

### Molecular docking

Molecular docking was performed between the screened target protein and tripterine. The three-dimensional (3D) structure of tripterine was obtained from PubChem (https://pubchem.ncbi.nlm.nih.gov/) and imported into AutoDock Tools 1.5.6 saved in the Protein Data Bank, Partial Charge (Q), and Atom Type (T) (PDBQT) format. The 3D structure of the target protein was obtained from the Protein Data Bank database (https://www.rcsb.org), and the water molecule and the original ligand were eliminated using PyMOL. Following this, the target protein was imported into AutoDock Tools 1.5.6 for hydrogenation, charge calculation, and non-polar hydrogen bonding, the results of which were saved in the PDBQT format. The size of the grid box was 40 × 40 × 40. Finally, molecular docking was performed using the CMD command of AutoDock Vina.

### Reagents and instruments

The following were the reagents and instruments used in this study: tripterine (Shanghai Yuanye Bio-Technology Co., Ltd, China); bleomycin (BLM) hydrochloride (Novartis Pharma AG, BASEL, Switzerland); anti-collagens I and III, anti-vimentin, and anti-α-smooth muscle actin (SMA) (Servicebio Technology Co., Ltd, China); anti-phosphatidylinositol-3-kinase (PI3K), anti-phospho-PI3K, anti-protein kinase B (Akt), and anti-phospho-Akt (Abmart, Shanghai, China) antibodies; anti-β-actin (Proteintech Group Inc., China); mouse tumour necrosis factor-α kit and mouse hydroxyproline kit (Nanjing Jiancheng Biotechnology Co., Ltd, China); terminal deoxynucleotidyl transferase-mediated deoxyuridine triphosphate nick end labelling (TUNEL) apoptosis assay kit (Beyotime Biotechnology Co., Ltd, China).

### Test animals and groups

Specific pathogen-free C57BL/6J male mice (18–20 g, 6–8 weeks old) were procured from Cytointelligen (Taizhou, China). All the mice were kept at room temperature and acclimatised for a week before initiating the experiment. All animal experiments involved in this study were approved by the Ethics Committee of the Nanjing University of Chinese Medicine. Thirty mice were divided into the following five groups: the normal, model, low-dose tripterine, medium-dose tripterine, and high-dose tripterine groups. The mice in the high-, moderate-, and low-dose groups were intraperitoneally injected with 2 mg/kg, 1 mg/kg, and 0.5 mg/kg tripterine, respectively, every other day. The same volume of normal saline was injected intraperitoneally in the mice belonging to the normal and model groups.

### Construction of the pulmonary fibrosis (PF) model

BLM intratracheal instillation was employed to simulate the CTD-ILD model. After the mice were anaesthetised with isoflurane in the supine position, the lumbar puncture needle was inserted into the trachea under light source irradiation. Following this, 50 μL of BLM solution was quickly injected into the trachea using a syringe. Finally, the mice were kept in an erect position and shaken to facilitate the even distribution of the solution in the lungs.

### Haematoxylin-eosin (H&E) and masson staining

Lung tissue was fixed in 4% paraformaldehyde and then dehydrated with alcohol at different concentration gradients. Subsequently, the sample was embedded in paraffin and sectioned for H&E and Masson staining. The degree of alveolitis and PF was scored according to the method reported by Ashcroft et al. (Ashcroft et al., 1988).

### Micro-computed tomography (CT) scanning

On the 28th day, the mice underwent micro-CT under isoflurane anaesthesia. Mouse lung imaging was performed using a Quantum GX Micro-CT scanner (PerkinElmer, Inc, Waltham, MA). Specific parameter settings are performed according to previous studies (Ruscitti et al., 2020).

### Measurement of hydroxyproline content in the lung tissue

The hydroxyproline level in the lung tissue samples was determined using a hydroxyproline assay kit. Twenty milligrams of lung tissue samples were homogenised using radioimmunoprecipitation assay (RIPA) buffer and proteinase inhibitors, and the supernatant was aspirated for use. The assay kit was removed from the refrigerator and kept at room temperature for at least 30 min. The concentrated wash buffer was diluted with double distilled water. The blank control, sample, and standard wells were set up on the plate according to the manufacturer’s instructions, and the corresponding reagents were added. A standard curve was plotted based on the measured optical density values, and the measured concentrations of the samples in each well were calculated based on the curve equation. These concentration values were then multiplied by the dilution factors to obtain the final concentrations.

### Measurement of TNF-α in the serum and lung homogenate

The samples were removed from the refrigerator (−80°C), and dissolved at room temperature. After the standard substances were diluted, the samples were loaded and washed, the enzyme was added, and the solution was incubated following the instructions of the TNF-α enzyme-linked immunosorbent assay kit. Following this, the linear regression equation was derived based on the absorbance value of the standard hole. The concentrations of the samples were calculated based on the absorbance values of the samples and the regression equation. Finally, the final concentration was obtained based on the product of the measured concentration and the dilution factor.

### Immunohistochemistry

Paraffin blocks were sectioned for dewaxing and hydration. To block the activity of the endogenous peroxidases for 10 min, 3% hydrogen peroxide was added. After antigenic repair, the paraffin blocks were incubated with serum blocking solution for 30 min to seal the non-specific binding site. Following this, the serum was removed, and the sections were washed. Subsequently, the sections were incubated overnight with the primary antibodies at 4°C. The next day, the slides were washed, treated with secondary antibodies, and stained with diaminobenzidine and haematoxylin. Brownish-yellow areas observed under light microscopy (×400 magnification) indicated a positive result.

### Western blot

The lung tissues were lysed using the RIPA buffer comprising proteinase inhibitors. The protein content was measured, separated using 10% sodium dodecyl sulphate-polyacrylamide gel electrophoresis, and then electroblotted onto a nitrocellulose membrane. Non-specific antigens were incubated in 5% bovine serum albumin with the membrane for 2 h. Following this, the membrane was incubated overnight with primary antibodies at 4°C. The membrane was washed thrice in 0.1% Tris-buffered saline with 0.1% Tween^®^ 20 Detergent (TBST) for 10 min at room temperature. The following day, the corresponding secondary antibody (1:2000) was added, and the mixture was incubated at room temperature for 1 h. The membrane was washed thrice with TBST three times for 10 min and then exposed to a colour-developing reagent under dark conditions. ImageJ 1.49 software was used to analyse the grey values.

### TUNEL staining of lung tissues

The paraffin section was dewaxed twice for 5–10 min. This was achieved by treating the section with anhydrous ethanol for 5 min, followed by 90% ethanol for 2 min, 70% ethanol for 2 min, and distilled water for 2 min. A drop of 20 μg/ml DNase-free protease K was added, and the section was kept at room temperature for 15–30 min. The section was then washed thrice with phosphate-buffered saline. Following this, the reaction solution was added separately. Finally, the sections were sealed using an antifade mounting medium with 4′,6-diamidino-2-phenylindole.

## Data analysis

Data analysis was performed using GraphPad Prism 8.0 software. Between-group differences were tested using one-way analysis of variance. Values are expressed as the mean ± standard deviation (* represents the difference between the BLM-treated and the other groups). Statistical significance was set as *p <* 0.05.

## Results

### Acquisition of the main intersection targets

**FIGURE 2:**
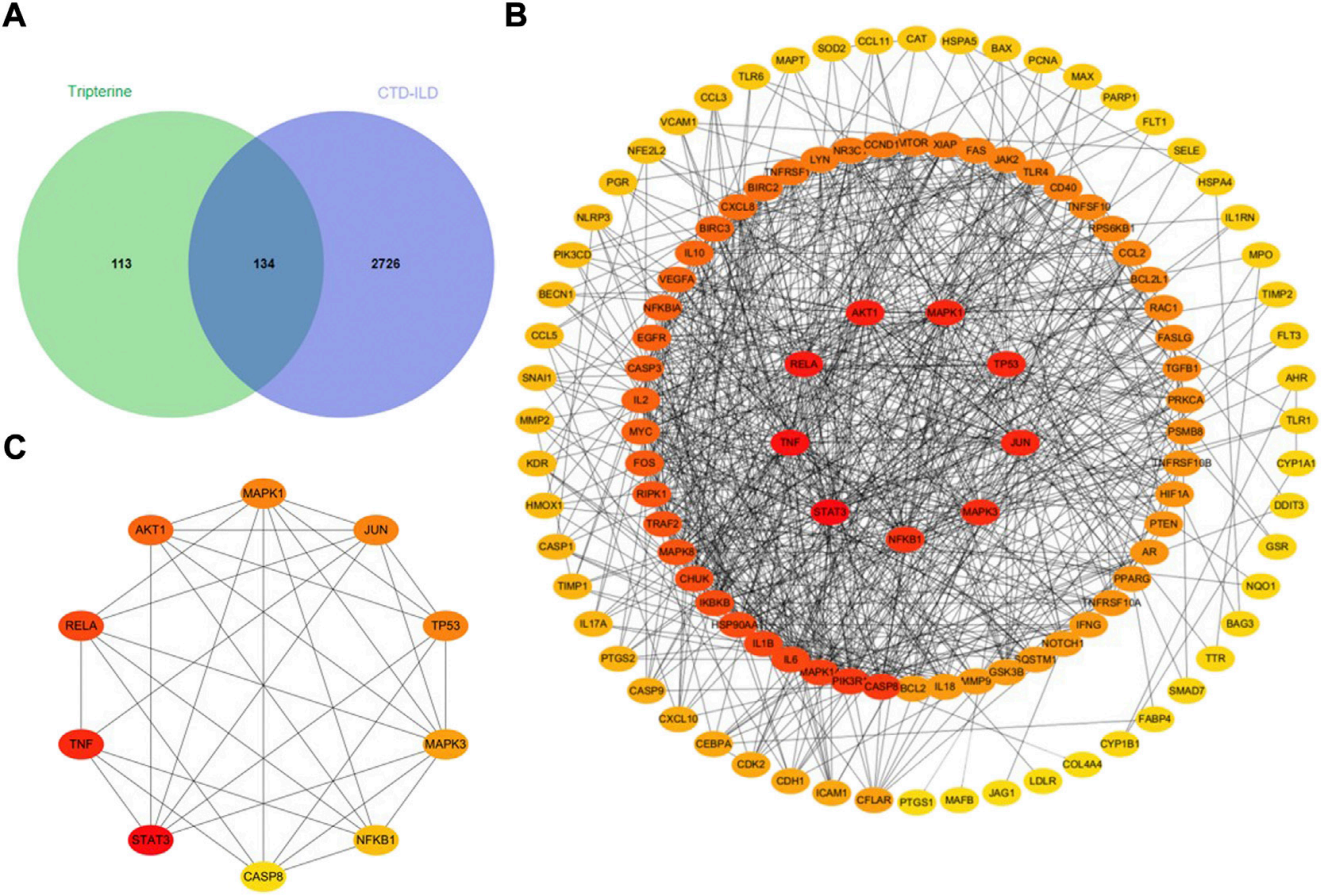
Analysis of the intersection targets of tripterine in the treatment of connective tissue disease-related interstitial lung disease (CTD-ILD). **(A)** A Venn diagram of the intersection targets between tripterine and CTD-ILD. **(B)** Topological analysis of the tripterine targets in the treatment of CTD-ILD. **(C)** Topological analysis of the top 10 targets of CTD-ILD treated with tripterine (different colours represent the varied importance in the network; red represents more importance, while yellow represents lesser importance).

A total of 247 tripterine targets were obtained by searching TCMSP, Comparative Toxicogenomics Database, GeneCards, STITCH, and SymMap databases. A total of 2,860 disease-related targets were obtained after eliminating duplicates by searching the OMIM, GeneCards, Genebank, and DrugBank databases. A total of 134 intersection targets were obtained by mapping disease-related targets to potential drug targets (Figure 2A).

### PPI network construction and topology analysis

A total of 134 target proteins were imported into the STRING 11.0 online database for protein correlation analysis, and the PPI network parameter of tripterine in the treatment of CTD-ILD was obtained. Subsequently, the screened PPI network was imported into Cytoscape 3.8.0 for topological analysis. The deeper the node colour, the higher the value, and the higher the core position of the target protein in the network (Figure 2B). Based on the results, the following were the top 10 targets: signal transducer and activator of transcription 3 (STAT3), TNF, v-rel avian reticuloendotheliosis viral oncogene homolog A (RELA), protein kinase B *α* (Akt1), mitogen-activated protein kinase (MAPK) 1, Jun transcription factor family (JUN), tumour protein 53 (TP53), MAPK3, nuclear factor kappa B subunit 1 (NF-κB1), and caspase 8 (CASP8) (Figure 2C). The details of the top 10 targets are presented in Table 1.

**TABLE 1:** The top 10 targets of tripterine in the treatment of CTD-ILD.

No.	Gene abbreviation	Betweenness centrality	Degree
1	STAT3	1597.11766	45
2	TNF	998.82458	41
3	RELA	723.37956	39
4	Akt1	1191.92445	36
5	MAPK1	1159.51724	34
6	JUN	825.73833	34
7	TP53	969.40455	34
8	MAPK3	428.44145	33
9	NF-κB1	458.3123	32
10	CASP8	555.13242	29

Abbreviations: STAT3, signal transducer and activator of transcription three; TNF, tumour necrosis factor; RELA, v-rel avian reticuloendotheliosis viral oncogene homolog A; Akt1, protein kinase B α; MAPK, mitogen-activated protein kinase; JUN, Jun transcription factor family; TP53, tumour protein 53; NF-κB1, nuclear factor kappa B subunit one; CASP8, caspase 8

### GO and KEGG enrichment analysis

**FIGURE 3:**
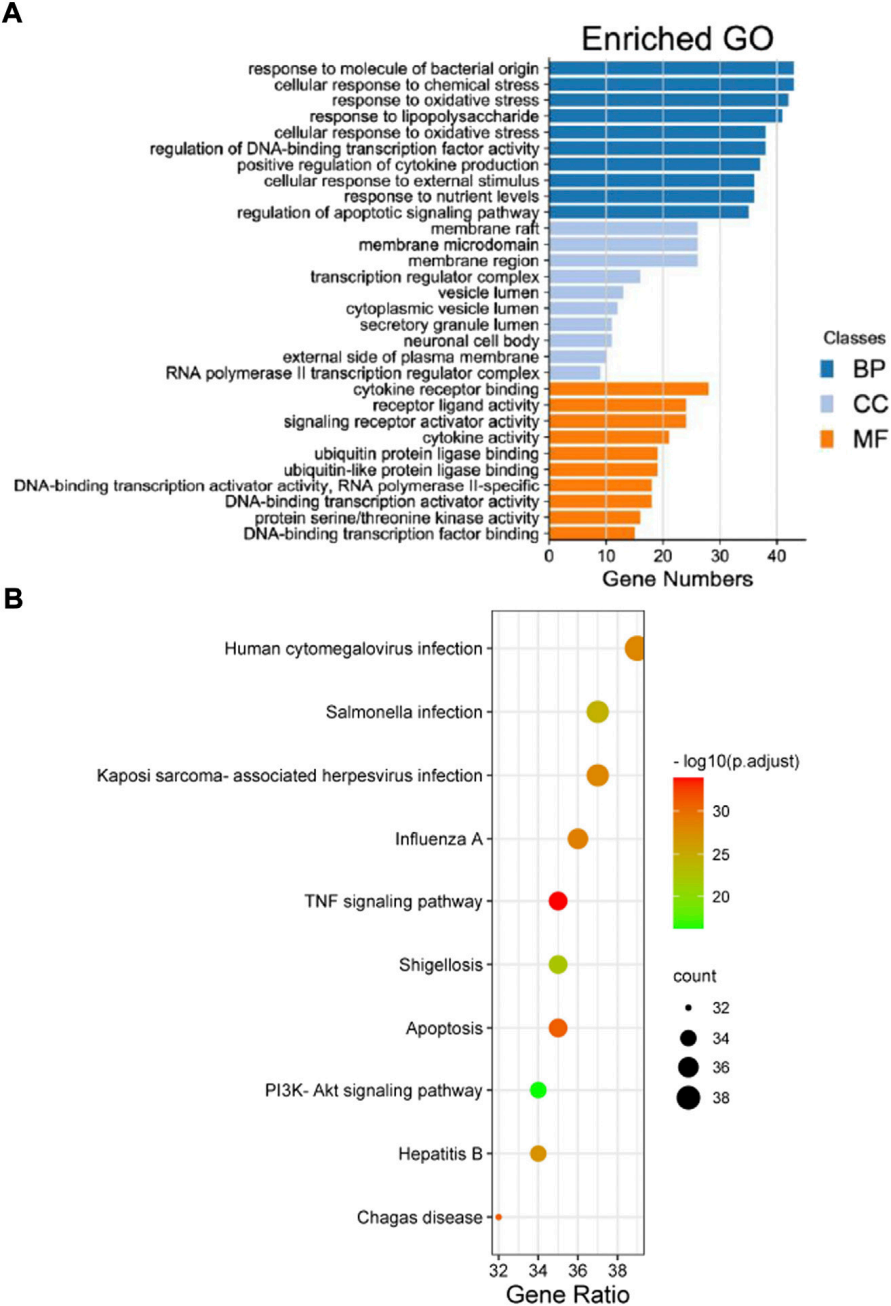
The Gene Ontology (GO) and Kyoto Encyclopaedia of Genes and Genomes (KEGG) pathway enrichment analyses results of the intersection targets. **(A)** The GO enrichment analysis identified genes involved in the GO-cellular component analysis, GO-biological process analysis, and GO-molecular function analysis. **(B)** The KEGG pathway analysis based on the bioinformatics data for the molecular signal pathway of tripterine against connective tissue disease-related interstitial lung disease.

A total of 2,719 items were obtained by the GO enrichment analysis. The first 10 items with *p*-values < 0.05 in the MF, BP, and CC categories were selected. The bubble size represented the number of targets during the GO analysis, and the bubble colour represented the enrichment significance. It mainly involved cytokine receptor binding, receptor-ligand activity, signal receptor activation, cytokine activity, protein ubiquitination, deoxyribonucleic acid transcriptase activity, ribonucleic acid transcriptase Ⅱ specificity, etc. (Figure 3A). A total of 155 items, including multiple viral infections and the PI3K/Akt, TNF, and apoptosis signalling pathways, were obtained from the KEGG pathway enrichment analysis. The first 30 related GO enrichment results and 10 KEGG enrichment analyses were visualised (Figure 3B).

### Molecular docking

**FIGURE 4:**
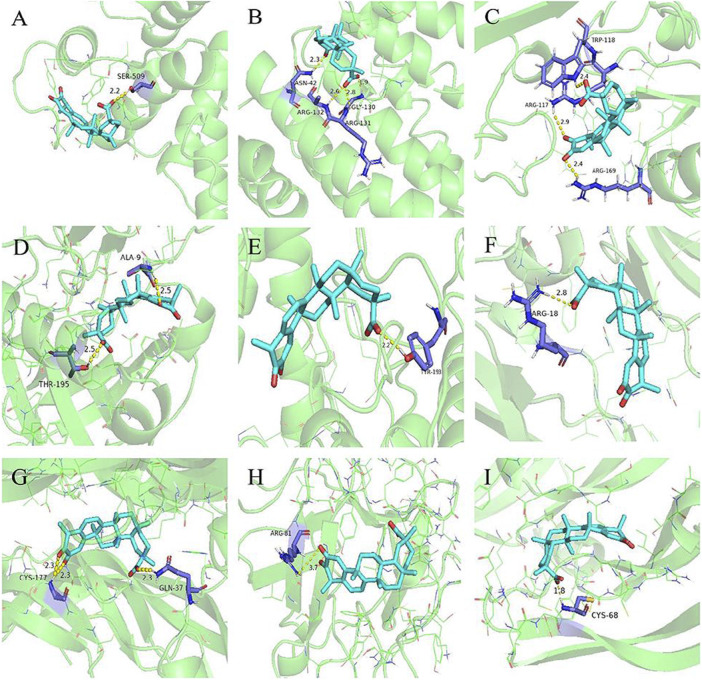
Molecular docking models of tripterine with the core targets. Proteins **(A)** signal transducer and activator of transcription 3 (6NUQ), **(B)** v-rel avian reticuloendotheliosis viral oncogene homolog A (6QHL), **(C)** tumour necrosis factor (3L9J), **(D)** protein kinase B *α* (6NPZ), **(E)** mitogen-activated protein kinase 1 (3W55), **(F)** transforming growth factor (3KFD), **(G)** platelet-derived growth factor (3MJK), **(H)** fibroblast growth factor (2K8R), **(I)** vascular endothelial growth factor (1VPF) are shown interacting with a tripterine molecule, represented by a blue stick model. Lines represent residues in the binding sites. The yellow dashed lines represent hydrogen bonds, and the interaction distances are indicated next to the bonds.

Molecular docking was performed between the first five core targets and tripterine. Furthermore, we docked tripterine with four main targets of nintedanib in the treatment of CTD-ILD. The docking results suggested that tripterine could exhibit good docking activities with target proteins such as STAT3, TNF, RELA, Akt1, MAPK1, TGF, PDGF, FGF, and VEGF (Figure 4). All binding energies were < −5 kcal/mol. In terms of molecular docking, the smaller the binding energy, the better the docking activity between ligand and receptor. Therefore, we reported that tripterine could interact with these core targets.

### Effects of tripterine on body weight and lung histopathology

**FIGURE 5:**
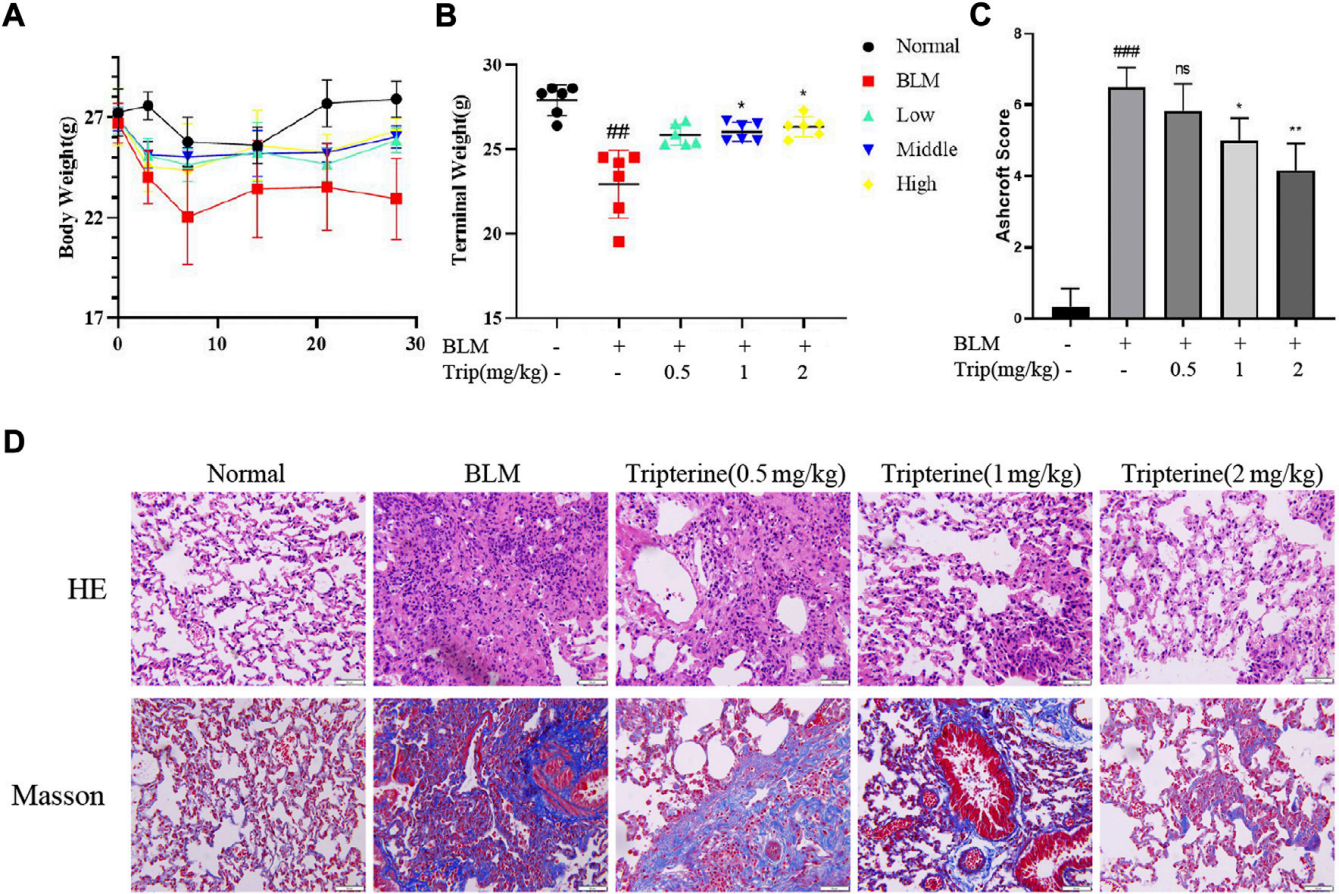
Changes in the body weight and lung pathology after tripterine treatment. **(A)** Changes in the body weight of the mice at different time points. **(B)** The endpoint weight of the mice in each group. **(C)** Ashcroft scores in a different group. **(D)** Haematoxylin-Eosin and Masson staining results of lung sections in a different group. Scale bars: 50 μm. Data are reported in the figures as the mean ± standard deviation, *n* = 6 in each group. ###*p* < 0.001 vs the normal group; *p < 0.05, **p < 0.01, and ***p < 0.001 vs. the BLM-treated group. BLM, bleomycin (5 mg/kg); Trip, tripterine.

After intratracheal instillation of BLM or normal saline, the mice lost weight during the first week. However, the mice belonging to the control group gained weight gradually after the second week. In contrast, the weight of the mice treated with tripterine belonging to the model group was relatively stable (Figure 5A). After 28 days of treatment, the endpoint weights of the mice in each group were evaluated. It was observed that the body weight of the mice in the BLM group significantly decreased, while the body weight of the mice receiving varying doses of tripterine was relatively stable (Figure 5B). H&E and Masson staining results in each group were analysed using the Ashcroft score. The result revealed that the mice treated with BLM had a significantly increased Ashcroft score (Figure 5C). Furthermore, the alveolar structure underwent destruction, and the alveolar cavity was infiltrated with inflammatory cells. However, after management with the administration of medium- or high-dose tripterine, the inflammatory cell infiltrate in the alveolar cavity significantly improved, and the alveolar structure underwent varying degrees of repair. Masson staining revealed that lung sections of the model group were filled with massive blue collagen deposits. The blue areas significantly decreased after the administration of tripterine (Figure 5D).

### Effects of tripterine on collagen deposition and Micro-CT

**FIGURE 6:**
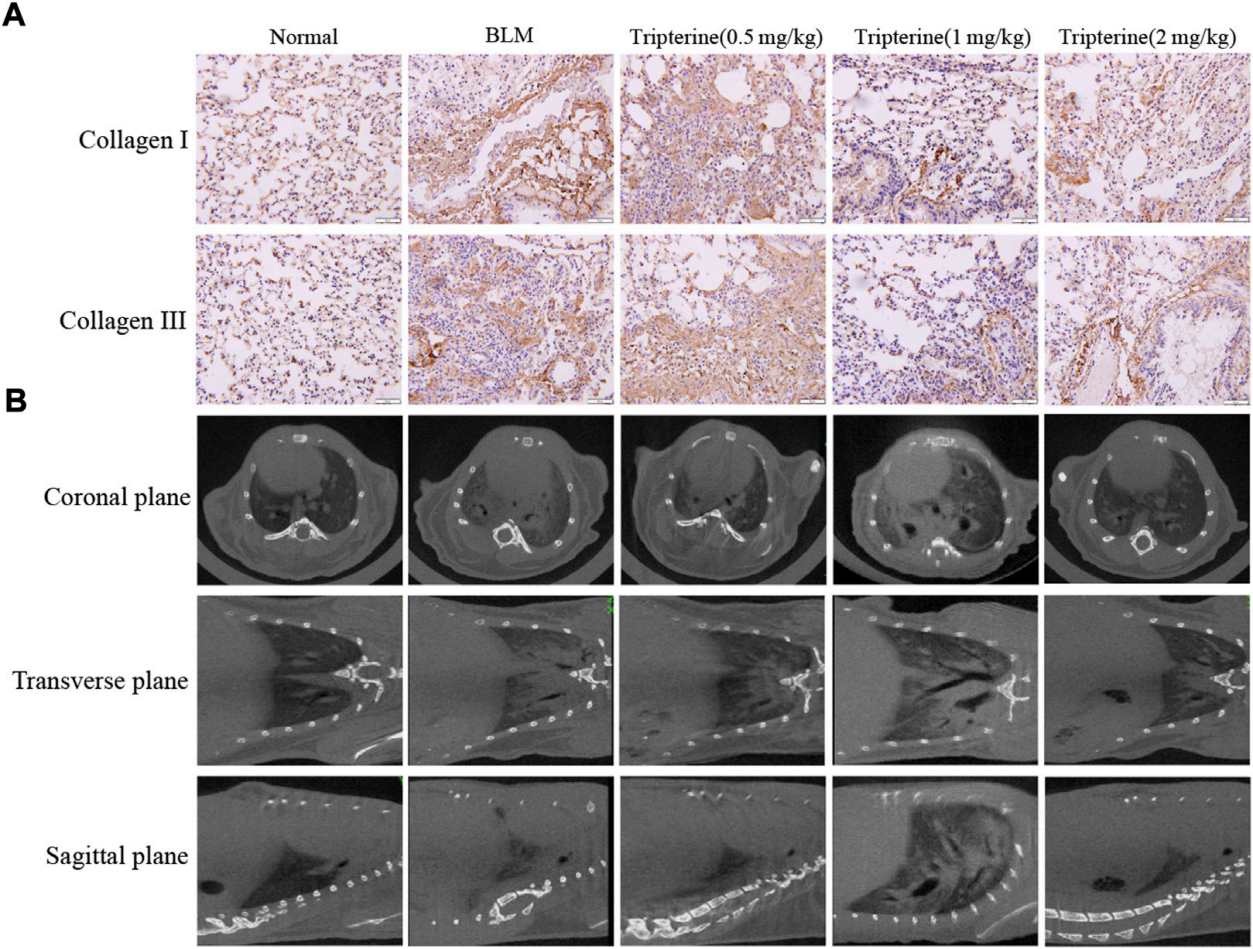
Deposition of collagen I and III and micro-computed tomography (CT) findings in mouse lungs. **(A)** Collagen I and III depositions in the lung tissues of mice after tripterine treatment. **(B)** Micro-CT findings of the lungs in mice treated with tripterine. Scale bars: 50 μm. Results are presented as the mean ± standard error of the mean. ###*p* < 0.001 vs the normal group; *p < 0.05, **p < 0.01, and ***p < 0.001 vs. the BLM-treated group. BLM, bleomycin (5 mg/kg); Trip, tripterine.

One of the main pathological manifestations as ILD progresses is the thickening of the alveolar septum caused by collagen deposition, which further affects lung diffusion. CT is widely used in the clinical diagnosis and management of ILD, and micro-CT is a mature tool for evaluating lung imaging changes in small animals (Ruscitti et al., 2018). Therefore, pulmonary collagen deposition and pulmonary micro-CT findings were evaluated in this study. After BLM was administered to the model mice, significant collagen I and collagen III deposits were observed in the lung tissue. Interestingly, collagen deposition was alleviated to varying degrees after varying doses of tripterine were administered (Figure 6A). Micro-CT results revealed typical lung consolidation in the model group. Lung consolidations significantly decreased after tripterine treatment, as observed on radiographs (Figure 6B).

### Effect of tripterine on the epithelial-mesenchymal transformation (EMT) marker protein in lung tissues

**FIGURE 7:**
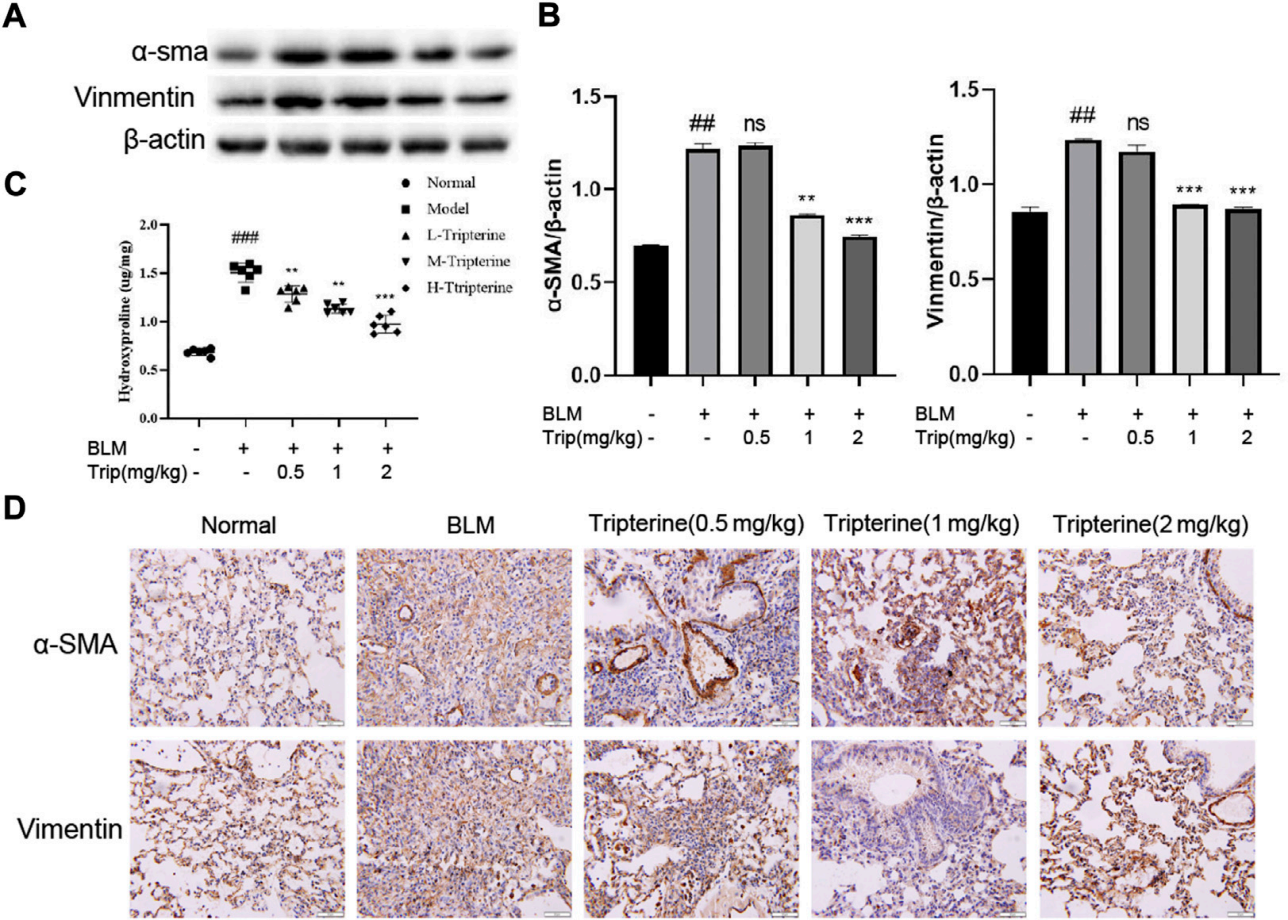
Effects of tripterine on epithelial-mesenchymal transformation markers of lung tissue in the connective tissue disease-related interstitial lung disease (CTD-ILD) model mice. **(A)** *α*-smooth muscle actin (SMA) and vimentin of lung tissues were analysed by Western blotting. **(B)** Expression changes in *α*-SMA and vimentin in the lung tissues of the mice were analysed by densitometry. **(C)** The hydroxyproline content in the lung tissues of the mice was determined by enzyme-linked immunosorbent assay. **(D)** Immunohistochemistry analysis of *α*-SMA and vimentin expression levels in the lung tissues of mice treated with tripterine. Scale bars: 50 μm. Data reported in the figures are presented as the mean ± standard deviation, *n* = 6 in each group. ###*p* < 0.001 vs. the normal group; *p < 0.05, **p < 0.01, and ***p < 0.001 vs. the BLM-treated group. BLM, bleomycin (5 mg/kg); Trip, tripterine.

α-SMA is a phenotypic marker of myofibroblasts in PF. Vimentin anchors and supports organelles in the mesenchymal cell cytoplasm. Both of these are involved in EMT (García-Cuellar et al., 2021). EMT-derived cells might also promote abnormal epithelial-mesenchymal crosstalk, thereby promoting fibre formation. Hydroxyproline is a non-essential amino acid found in collagen and is one of its main components. It is also an important marker of PF. Herein, we found that the *α*-SMA, vimentin, and hydroxyproline expression levels significantly increased in the model group and significantly decreased after 28 days of treatment with tripterine, particularly in the medium- and high-dose groups (Figures 7A–D).

### Effects of tripterine on the PI3K/Akt and TNF-α signalling pathways

**FIGURE 8:**
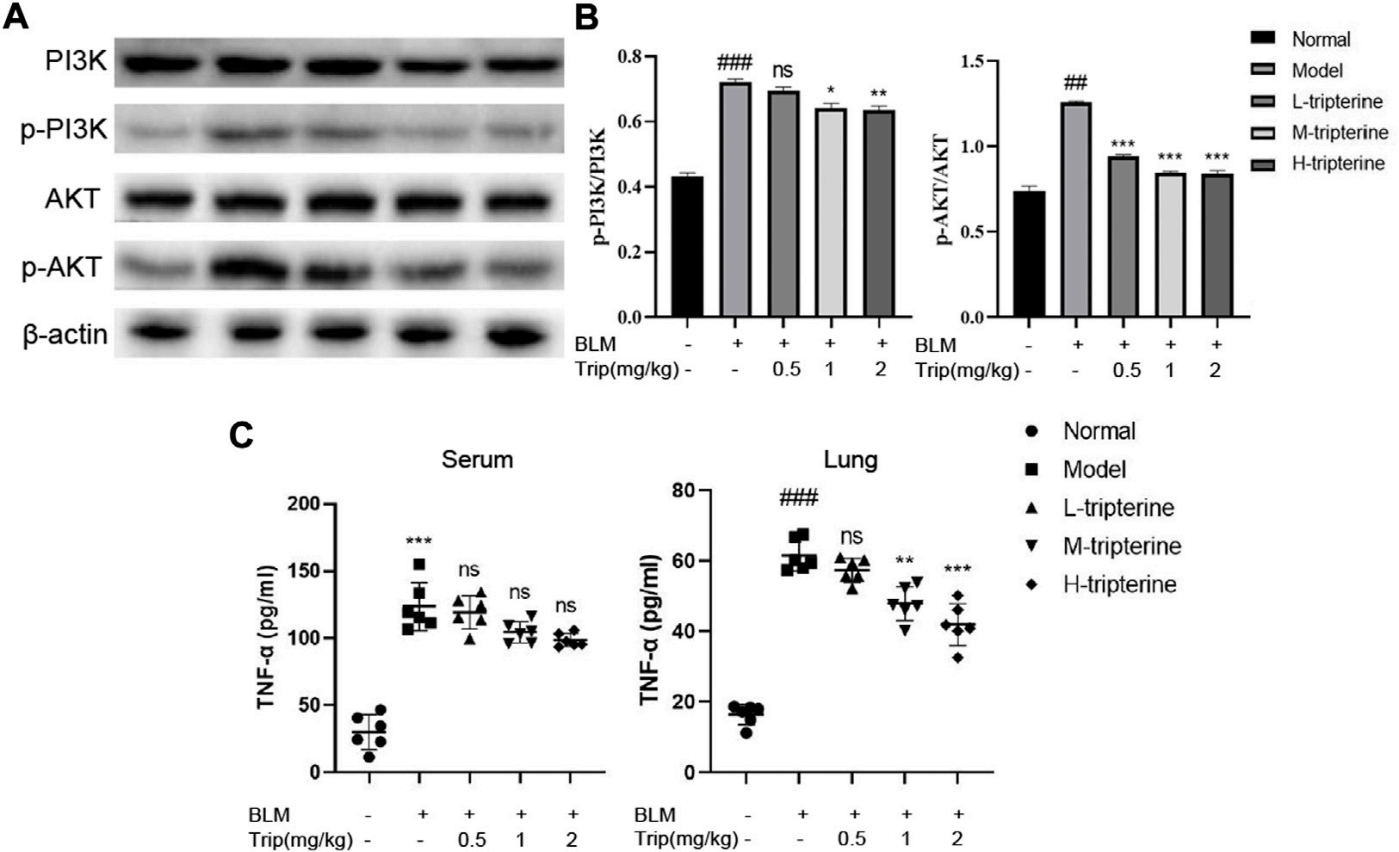
Effects of tripterine on phosphatidylinositol-3-kinase (PI3K)/protein kinase B (Akt) and tumour necrosis factor (TNF)-α expression levels. **(A)** PI3K/Akt-related protein expression in lung tissues was detected by Western blotting. **(B)** Densitometry analysis of PI3K/Akt protein expression. Scale bars: 50 μm. Data are presented as the mean ± standard deviation (SD), *n* = 3. **(C)** TNF-α expression in the lung homogenate and serum of mice were detected by enzyme-linked immunosorbent assay. Data are presented as the mean ± SD, *n* = 6. ###*p* < 0.001 vs. the normal group; *p < 0.05, **p < 0.01, and ***p < 0.001 vs. the BLM-treated group. BLM, bleomycin (5 mg/kg); Trip, tripterine.

Our network pharmacology study reported that the PI3K/Akt and TNF-α signalling pathways might be involved in the anti-CTD-ILD effect of tripterine. Therefore, we measured the key proteins of the PI3K/Akt signalling pathway and the TNF-α content in the serum and lung tissues to identify the effects of tripterine on these pathways. Our results revealed that the phosphorylated PI3K and Akt levels significantly increased in the model group but decreased to varying degrees after tripterine administration (Figures 8A,B). Similarly, TNF-α levels increased in the model group and decreased in the lung tissue of the model mice after tripterine administration. However, no significant improvement was observed in the serum TNF-α levels of the model mice (Figure 8C).

### Effect of tripterine on the apoptosis level of lung tissues

**FIGURE 9:**
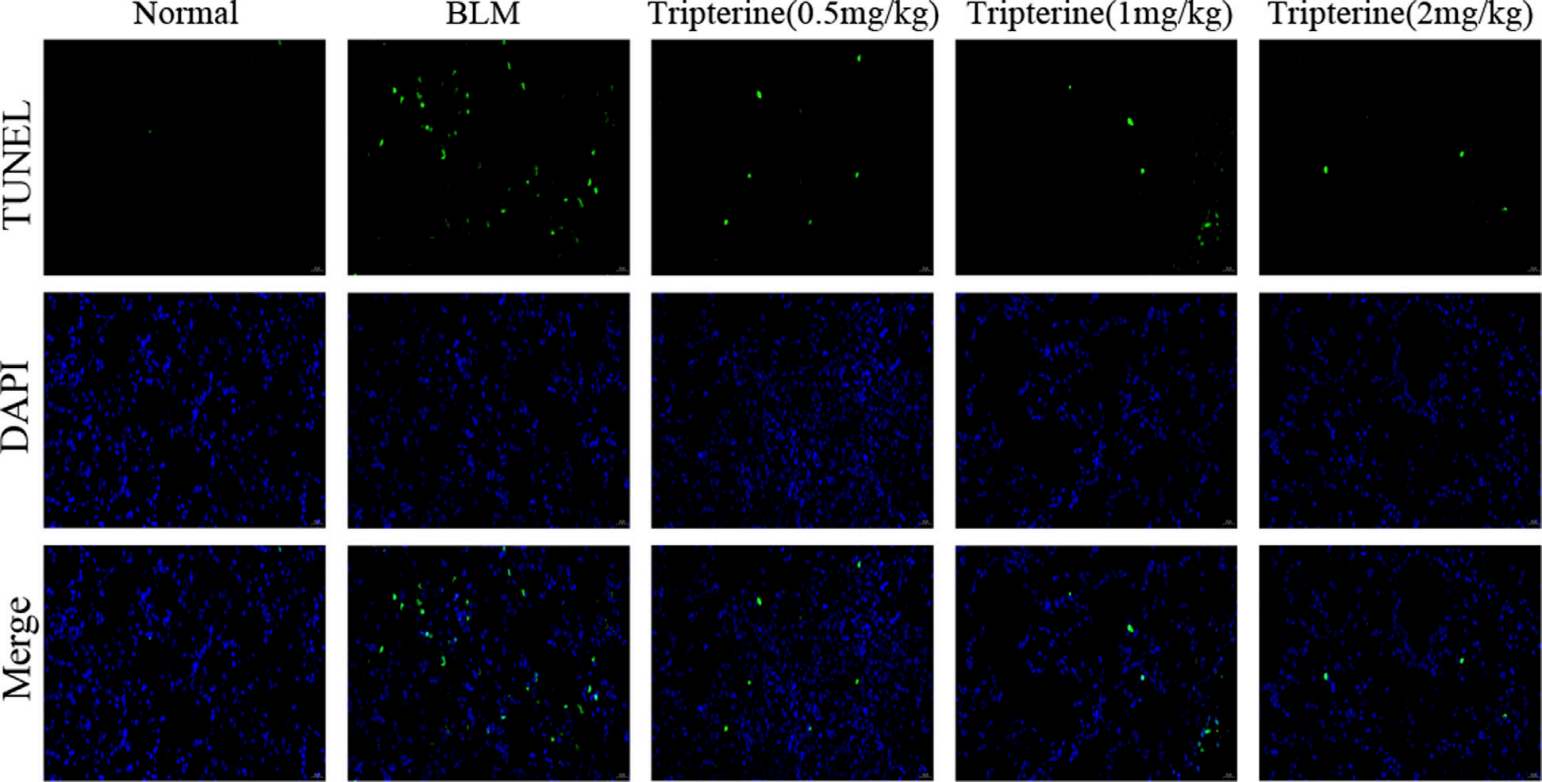
The apoptosis level of the lung tissues was analysed by immunofluorescence. Normal: normal group; BLM, bleomycin (5 mg/kg); scale bars: 100 μm.

Apoptosis is a biological process closely associated with ILD (Nho et al., 2022). The apoptosis level of the lung tissues in each group was observed by immunofluorescence assay to validate the results of our KEGG analysis. Apoptotic cells significantly increased in the lung tissues of the model mice, based on the immunofluorescence assay results. We observed that the positive rate of apoptotic staining in the lung tissues decreased gradually with an increase in the tripterine dose (Figure 9).

## Discussion

CTD-ILD is a heterogeneous disease characterised by diffuse alveolar inflammation and fibrosis (Atzeni et al., 2018), with progressive aggravation of clinical symptoms, ultimately leading to respiratory failure, high mortality, and limited therapeutic options. Due to the heterogeneity and interdisciplinary nature of ILD, a high-level scientific basis for this disease is scarce (Mira-Avendano et al., 2019; Johannson et al., 2021). It also lacks treatment and monitoring guidelines or an expert consensus. In the development and evolution of this disease, Immune imbalance is the main pathological factor responsible for the development and progression of ILD, and controlling the various pro-inflammatory factors and the restoration of immune homeostasis help inhibit the progression from inflammation to fibrosis in ILD (Kolahian et al., 2016). Currently, it is believed that the damage-repair process along with epithelial cell injury (Chambers and Mercer, 2015), innate and adaptive immunity (Kolahian et al., 2016; Desai et al., 2018), and humoral factors (De Lauretis et al., 2013; Wu et al., 2017) are involved in the process of fibrosis. Hence, Anti-inflammatory and antifibrotic therapy are the hot spots in the treatment of this disease (Wells, 2021). Studies have demonstrated that tripterine could decrease lung tissue injury by reducing capillary permeability, inhibiting inflammatory cytokines or chemokines, and governing the expression of numerous inflammatory mediators (Law et al., 2011). The pathogenesis of PF is typically characterised by excessive extracellular matrix (ECM) deposition and EMT, during which several factors change, including excessive accumulation of type I and type III collagens, the main components of the ECM, and the up-regulation of mesenchymal markers, such as *α*-SMA and vimentin (Hsieh et al., 2022). Through *in vivo* experiments, we reported that tripterine could decrease lung pathology and collagen deposition in the lung tissues of the model mice. Increased levels of fibrosis markers, namely *α*-SMA and vimentin, were observed in the model group. The levels of these proteins in the lung tissue decreased to varying degrees after treatment with tripterine. These results suggest that tripterine could reduce BLM-induced fibrosis in model mice. Therefore, we used network pharmacology to investigate the related targets and pathways of tripterine in the treatment of CTD-ILD.

A total of 134 potential targets of tripterine were obtained for treating CTD-ILD, of which STAT3, TNF, RELA, Akt1, MAPK1, JUN, TP53, MAPK3, NF-κB1, and CASP8 were strongly correlated with CTD-ILD. Additionally, the above-mentioned core targets were closely associated with PF. KEGG pathway analysis revealed that the potential mechanisms of triptolide in the treatment of CTD-ILD might be attributed to multiple viral infections and the TNF, PI3K/Akt, and apoptosis signalling pathways. These pathways are involved in the pathogenesis of various inflammatory diseases and are crucial for anti-pulmonary fibrosis progression (Meng et al., 2014).

Firstly, viruses have been considered pathogens or propagators of ILD-related inflammation (Wootton et al., 2011), and multiple viral infections can significantly increase the risk of interstitial pneumonia (Sheng et al., 2020). Recent studies (Pehote and Vij, 2020) have reported that viral infection impedes autophagosome formation in patients with ILD, and autophagy function was damaged due to invasive accumulation, thereby promoting the senescence of lung epithelial cells and myofibroblast differentiation.

Secondly, according to the KEGG enrichment analysis results, apoptosis, TNF-α, and PI3K/Akt signalling pathways might be related to the mechanism of tripterine in CTD-ILD treatment, as verified by *in vivo* experiments. The TNF signalling pathway is a pro-inflammatory signalling pathway that plays a crucial role in joint and lung inflammatory diseases. Previous studies (Wu et al., 2019) have reported that anti-TNF treatment could selectively increase the monocyte and dendritic cell count in lung tissues, thereby achieving the therapeutic effect of alleviating joint and lung inflammation. Furthermore, fibroblast to myofibroblast transformation, which is considered to play a crucial role in CTD-ILD pathogenesis, can be enhanced by TNF-α (Spagnolo et al., 2021). Apoptosis is a key mechanism for regulating cell death (Sauler et al., 2019), which is beneficial for eliminating damaged, infected, or excessive cells and is associated with the occurrence of ILD (Mahavadi et al., 2010). We reported that tripterine could reduce the fluorescence intensity of TUNEL staining in lung tissues, indicating that it could reduce the occurrence of apoptosis in the lung tissues of model mice. Furthermore, a significant increase in TNF-α levels was observed in the model group. TNF-α levels in lung tissues significantly decreased after tripterine administration.

In addition, the PI3K/Akt signalling pathway has been confirmed to play a pathogenic role in ILD. For example, TGF-β can promote EMT, induce fibrosis through the PI3K/Akt signalling pathway, and modulate fibroblast differentiation into myofibroblasts that regulate ECM accumulation (Huang et al., 2021). PI3K is considered to be the focus of collagen synthesis induced by most pathways, and currently, a non-targeting PI3K protein inhibitor for treating idiopathic pulmonary fibrosis is undergoing human clinical trials (Hettiarachchi et al., 2020). Akt is the downstream protein of PI3K. Blocking these protein targets also has a corresponding effect on inhibiting PF progression. Our experiment demonstrated that tripterine could inhibit the activation of the PI3K/Akt signalling pathway in the lung tissues of model mice.

These results were consistent with our predictions based on network pharmacology analysis. Our findings suggested that tripterine attenuates collagen accumulation and fibrosis in lung tissue, resulting in the inhibition of the PI3K/Akt, apoptosis, and TNF-α signalling pathways. This may partly explain why a lesser degree of inflammation and fibrosis were observed in the BLM-induced lungs. Indubitably, this study has some limitations, which need to be resolved by further research. First, various cells play an important role in the pathological development of ILD; however, specific cellular experiments were not performed to verify the above findings in this study. Secondly, whether the PI3K/Akt signalling pathway plays a role through the apoptotic pathway and the concrete downstream regulatory mechanism involved needs to be elucidated. Lastly, the BLM-induced ILD model undoubtedly does not completely mimic immune-mediated lung interstitial lesions, and whether tripterine has the same effect in ILD models induced by other factors likewise needs to be explored.

## Conclusion

Tripterine can lower the degree of pathology and fibrosis in BLM-induced ILD in mice. The specific mechanism may be related to inhibiting the PI3K/Akt, apoptosis, and TNF-α signalling pathways. *Tripterygium wilfordii* Hook. F. and its extract can be employed clinically for treating CTD-ILD.

## Data Availability

The original contributions presented in the study are included in the article/Supplementary Material, further inquiries can be directed to the corresponding author.

## Abbreviations

3D, three-dimensional; *α*-SMA, *α*-smooth muscle actin; Akt, protein kinase B; Akt1, protein kinase B α; BLM, bleomycin; BP, biological process; CASP8, caspase 8; CC, cellular components; CT, computed tomography; CTD, connective tissue disease; CTD-ILD, connective tissue disease-related interstitial lung disease; ECM, extracellular matrix; EMT, epithelial-mesenchymal transition; FGF, fibroblast growth factor; GO, Gene Ontology; H&E, haematoxylin-eosin; ILD, interstitial lung disease; JUN, Jun transcription factor family; KEGG, Kyoto Encyclopaedia of Genes and Genomes; MAPK, mitogen-activated protein kinase; MF, molecular function; NF-κB1, nuclear factor kappa B subunit 1; OMIM, Online Mendelian Inheritance in Man; PDBQT, Protein Data Bank, Partial Charge (Q), and Atom Type (T); PDGF, platelet-derived growth factor; PF, pulmonary fibrosis; PI3K, phosphatidylinositol-3-kinase; PPI, protein-protein interaction; RELA, v-rel avian reticuloendotheliosis viral oncogene homolog A; RIPA, radioimmunoprecipitation assay; STAT3, signal transducer and activator of transcription 3; STITCH, Search Tool for Interacting Chemicals; TBST, Tris-buffered saline with 0.1% Tween^®^ 20 Detergent; TCMSP, Traditional Chinese Medicine System Pharmacology Database and Analysis Platform; TGF, transforming growth factor; TNF, tumour necrosis factor; TP53, tumour protein 53; TUNEL, terminal deoxynucleotidyl transferase-mediated deoxyuridine triphosphate nick end labelling; VEGF, vascular endothelial growth factor.
